# Hetrombopag plus porcine ATG and cyclosporine for the treatment of aplastic anaemia: early outcomes of a prospective pilot study

**DOI:** 10.1186/s40164-023-00377-3

**Published:** 2023-02-01

**Authors:** Wenrui Yang, Xin Zhao, Xu Liu, Youzhen Xiong, Huihui Fan, Li Zhang, Jianping Li, Lei Ye, Kang Zhou, Yuan Li, Yang Yang, Guangxin Peng, Liping Jing, Fengkui Zhang

**Affiliations:** State Key Laboratory of Experimental Hematology, Haihe Laboratory of Cell Ecosystem, National Clinical Research Center for Blood Diseases, Institute of Hematology & Blood Diseases Hospital, Chinese Academy of Medical Sciences & Peking Union Medical College, 288 Nanjing Road, Heping District, Tianjin, 300020 China

**Keywords:** Hetrombopag, Aplastic anaemia, Immunosuppressive therapy

## Abstract

Hetrombopag, a small molecular thrombopoietin-receptor agonist, has shown encouraging efficiency in immunosuppressive therapy refractory or relapsed severe aplastic anaemia. To investigate the response rate of hetrombopag combined with IST as first-line treatment, we designed a prospective pilot study including 32 patients with SAA treated with anti-human T lymphocyte porcine immunoglobulin (p-ATG), cyclosporine, and hetrombopag. In addition, 96 patients with SAA treated with p-ATG and cyclosporine alone were matched as controls. In total, 21.9% of patients treated with hetrombopag achieved complete response (CR) at 3 months, while 5.2% of patients achieved CR in the control group (*P* = 0.005). At 6 months, the CR rates were 34.4% in the hetrombopag group and 14.6% in the control group (*P* = 0.015). The overall response rates at 6 months were 68.7% and 50.0% in the hetrombopag and control groups, respectively. The median time to haematologic response was 56 days and 77 days, and to CR was 96 days and 214 days in the hetrombopag and control groups, respectively. In conclusion, adding hetrombopag to IST as first-line treatment resulted in faster and better haematologic response in SAA.

**Supplementary Information:**

The online version contains supplementary material available at 10.1186/s40164-023-00377-3.

## To the editor,

Aplastic anaemia (AA), a bone marrow failure disease, develops from T cell-mediated haematopoietic stem cell destruction [1]. Its response rate to standard immunosuppressive therapy (IST) is 60–70%, and the long-term overall survival is approximately 80% [1, 2]. Previously, improving the efficacy of IST was a challenge until its co-administration with eltrombopag (EPAG) was investigated. EPAG, an oral non-peptide thrombopoietin-receptor agonist (TPO-RA), was initially used in refractory severe AA (SAA) and showed an unexpected response rate of 40–50% [3, 4]. Consequently, two prospective studies showed earlier complete response (CR) and higher CR rates when EPAG plus standard IST was used in treatment-naïve SAAs [5, 6]. Currently, standard IST plus EPAG is the first-line choice for patients with SAA who are ineligible for haematopoietic stem cell transplantation (HSCT) [7].

Hetrombopag (HPAG), another oral non-peptide TPO-RA, has superior efficacy to EPAG [8]. In an open-label, non-randomized, prospective study, HPAG showed an overall response of 40% in IST-refractory SAAs [9]. It was approved for IST-refractory SAA by the China Food and Drug Administration in 2021 [10]. However, the efficacy of HPAG plus IST as first-line treatment for SAA is currently unclear; therefore, herein, we investigated this regimen at the Institute of Hematology & Blood Diseases Hospital, Chinese Academy of Medical Sciences. The methods of this study were described in Additional file 1.

### Patient characteristics

The median age in the HPAG group was 44 (13–69) years, with 17 males and 15 females. The median follow-up time was 366 (295–449) days in the HPAG group. The time of the last follow-up was > 12 months in the control group. Patient clinical characteristics are listed in Table 1.

**Table 1:** Clinical characteristics at baseline

	Hetrombopag group (n = 32)	Control group (n = 96)	*P* value
Age (years, median, range)	44 (13–69)	45 (7–70)	0.928
Gender (male/female)	17/15	52/44	0.919
Severity of aplastic anaemia, n (%)			
Severe	21 (66%)	60 (63%)	0.752
Very severe	11 (34%)	36 (37%)	
Complete blood count (median, range)			
Reticulocyte count (×10^9^/L)	15.2 (0.2–68.4)	12.9 (0–63.7)	0.821
Neutrophil count (×10^9^/L)	0.33 (0–1.04)	0.33 (0–1.12)	0.739
Platelet count (×10^9^/L)	7 (1–21)	8 (0–31)	0.361
Haemoglobin level (g/L)	55 (34–99)	61 (33–96)	0.046
PNH clones (+), n (%)	11 (34%)	26 (27%)	0.433

Hetrombopag group: IST plus hetrombopag; control group: IST alone

### Haematologic response

**Fig. 1:**
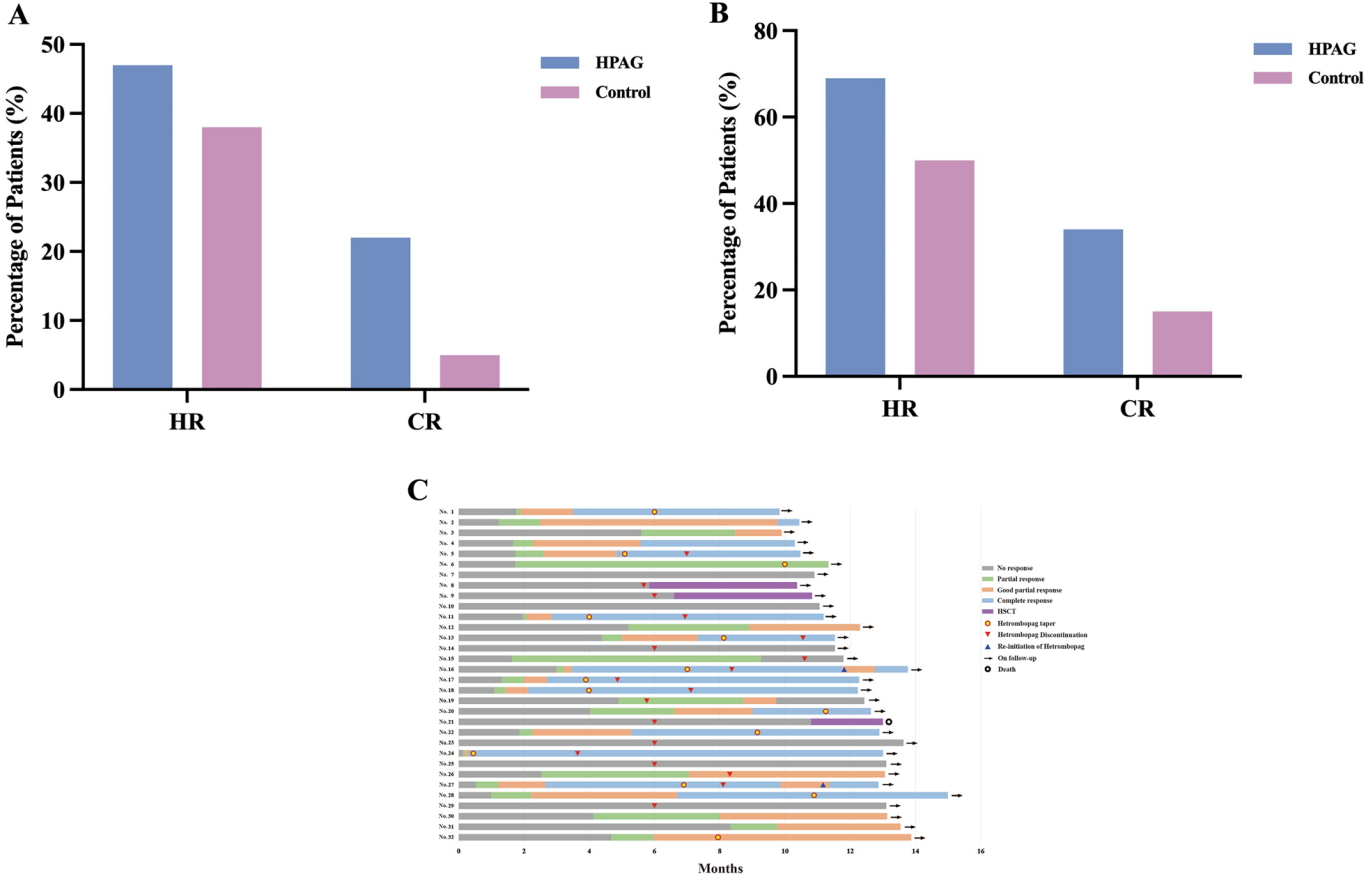
Graph A shows that the HR and CR rates at 3 months were 46.9% vs. 37.5% and 21.9% vs. 5.2%, respectively, in the two groups. Graph B shows that the HR and CR rates at 6 months were 68.7% vs. 50.0% and 34.4% vs. 14.6%, respectively, in the two groups. Graph C shows the haematologic responses over time in each patient. In total, 23 patients had a response after hetrombopag with IST treatment. However, patients No. 15 and No. 19 had disease relapse, one owing to discontinued hetrombopag, and the reason was unknown for the other. Patients No. 16 and No. 27 had fluctuating platelet counts of < 100 × 10^9^/L after discontinuing hetrombopag; however, their platelet counts returned to normal after re-initiation of hetrombopag at a dose of 7.5 mg. Only one patient died during HSCT at 393 days after IST. *HR* haematologic response, *CR* complete response

At 3 months, the OR rates were 46.9% and 37.5% in the HPAG and control groups, respectively (*P* = 0.350). The CR rate was 21.9% in the HPAG group and 5.2% in the control group (*P* = 0.005) (Fig. 1A). At 6 months, the OR rates were 68.7% and 50.0% in the HPAG and control groups, respectively (*P* = 0.066). The CR rate was 34.4% in the HPAG group and 14.6% in the control group (*P* = 0.015) (Fig. 1B). For disease severity, a higher CR rate was observed at 3 months (23.8% vs. 6.7%) and 6 months (42.9% vs. 16.7%) in patients with SAA who received HPAG. However, the OR rate was similar between both groups. HPAG addition did not affect the OR and CR rates in patients with VSAA (Additional file 1: Table S1).

### Time to response

The median time to first response was 56 days in the HPAG group and 77 days in the control group (*P* = 0.000) (Additional file 1: Fig. S1A). The median time to CR was 96 days in the HPAG group and 214 days in the control group (*P* = 0.019) (Additional file 1: Fig. S1B). The median time to achieving platelet counts of 100 × 10^9^/L was 88 days in the HPAG group and 207 days in the control group (*P* = 0.030).

### Safety and follow-up

HPAG was well tolerated, and no patient discontinued therapy because of side effects. Seven patients discontinued HPAG owing to no response at 6 months, and eight stopped owing to platelet counts. One patient had a relapse of AA after discontinuing HPAG. One patient had disease relapse during therapy and HPAG was stopped. HPAG was tapered in five patients and maintained at a dose of 15 mg in eight patients. Platelet counts decreased to < 100 × 10^9^/L in two patients with CR who tapered HPAG to discontinuation. Nonetheless, they re-achieved CR after re-initiating treatment at a dose of 7.5 mg (Fig. 1C).

By the last follow-up, the OR and CR rates were 65% and 43.8%, respectively. Two patients had a relapse of AA, and no patient died within 6 months after IST in the HPAG group. However, one patient died during the HSCT course (Fig. 1C); there were no complications, including haemolytic paroxysmal nocturnal haemoglobinuria and myelodysplastic syndromes/acute myeloid leukemia.

### Discussion

EPAG plus standard IST has been shown to improve response rate and quality in SAA [5, 6]. HPAG has comparable efficacy as EPAG in IST-refractory SAA [9]. Therefore, we conducted this study to determine its use as a first-line treatment. HPAG plus IST significantly increased the CR rate. The HR rate increased by approximately 10% and 20% in the HPAG group compared to the control group at 3 and 6 months, respectively. Nevertheless, the difference was not significant owing to the small sample size. HPAG plus IST improved haematologic and CR rates in patients with SAA or VSAA, especially the CR rate in patients with SAA, which is consistent with RACE study results.

The National Institutes of Health study showed a cumulative relapse rate of 39% in patients with SAA treated with IST plus EPAG, compared to IST alone [11]. Herein, two patients experienced relapse which may be associated with dose tapering of cyclosporine and HPAG. The median follow-up time of this study was relatively short; thus, we could only determine the early outcomes. Further follow-up is needed to analyse long-term outcomes. This is just a pilot study, with a prospective, randomized, double-blind, placebo-control clinical trial (NCT04961710) ongoing in China to confirm these results.

HPAG plus IST, compared with IST alone, was beneficial in patients with SAA and induced a higher quality and faster haematologic response without increasing adverse events.

## Supplementary Information

**Additional file 1.** The details of the methods, including study design, treatment protocol, haematologic response criteria, and statistical analysis. The predicting factors for haematologic response for IST plus hetrombopag. Table S1 showed haematologic response according to severity of aplastic anemia.

## Data Availability

The datasets used and/or analyzed during the current study are available from the corresponding author on reasonable request.

## Abbreviations

AA: Aplastic anaemia
IST: Immunosuppressive therapy
EPAG: Eltrombopag
HPAG: Hetrombopag
SAA: Severe aplastic anaemia
TPO-RA: Thrombopoietin-receptor agonist
CR: Complete response
HSCT: Haematopoietic stem cell transplantation
VSAA: Very severe aplastic anaemia
PR: Partial response
OR: Overall response
