# Supplementary material for: Hetrombopag plus porcine ATG and cyclosporine for the treatment of aplastic anaemia: early outcomes of a prospective pilot study
Source: Exp Hematol Oncol. 2023 Feb 1;12:16. doi: 10.1186/s40164-023-00377-3 (PMC9890734; doi:10.1186/s40164-023-00377-3)
Supplement: Supplementary file 1 — Additional file 1. The details of the methods, including study design, treatment protocol, haematologic response criteria, and statistical analysis. The predicting factors for haematologic response for IST plus hetrombopag. Table S1 showed haematologic response according to severity of aplastic anemia. [file 40164_2023_377_MOESM1_ESM.docx]

**Additional file 1**

**Methods**

***Study design***

This prospective single-arm pilot study of HPAG with porcine antithymocyte globulin (p-ATG) and cyclosporine (CsA) in acquired AA was conducted in the Anemia Therapeutic Center of the Institute of Hematology & Blood Diseases Hospital, Chinese Academy of Medical Sciences. A total of 32 patients who were diagnosed with SAA, VSAA, or transfusion-dependent non-severe aplastic anemia were enrolled for the study between August and December 2021. Patients were retrospectively compared to matched patients with AA previously treated with p-ATG and CsA alone between 2017 and 2019 in our department. Matching was performed in a 1:3 ratio using the nearest neighbour in SPSS software with a calliper width of 0.20. Patients were matched by age and disease severity (non-VSAA and VSAA) (32 patients treated with IST plus HPAG and 96 historical controls).

***Treatment***

HPAG was administered orally at a dose of 15 mg daily from day 1 of p-ATG treatment and maintained for 6 months. Within 6 months after ATG initiation, if the patient achieved CR with a platelet count of between 200–400×10^9^/L, HPAG was tapered at 2.5 mg per 2 weeks until platelet count was < 200×10^9^/L. If platelet count was > 400×10^9^/L, HPAG was discontinued until platelet count reached ≤ 200×10^9^/L and then restarted at a dose of 12.5 mg daily. HPAG was withdrawn if the platelet count remained > 400×10^9^/L at the lowest dosage. After 6 months, HPAG was tapered at 2.5 mg per month in patients who achieved CR or maintained the best haematologic response for 3 months. HPAG was withdrawn at 6 months for non-responders.

Anti-human T lymphocyte immunoglobulin (p-ATG, Yujin Bio-Pharma Wuhan CNBG Co., Ltd., Wuhan, Hubei, China) was administered for five consecutive days at a dose of 20 mg/kg/day. CsA was administered orally in divided doses and adjusted to maintain a serum trough concentration and peak concentration between 150–250 ng/mL and 800–1000 ng/mL, respectively. CsA was administered for at least 6 months. Among responders, CsA was slowly tapered at 50 mg every 3 months after achieving the best haematologic response.

Red blood cell (RBC) transfusion was administered to maintain a haemoglobin (HGB) level > 70 g/L. Platelets were transfused if the platelet count was < 10×10^9^/L or < 20×10^9^/L if the patient had fever or sepsis. Granulocyte colony-stimulating factor (G-CSF) was administered at a dose of 5–10 μg/kg when absolute neutrophil count (ANC) was < 0.5×10^9^/L.

***Haematologic response criteria***

CR was defined as haemoglobin > 100 g/L, ANC > 1.0×10^9^/L, and platelet count > 100×10^9^/L. Partial response (PR) was defined as transfusion independence, haemoglobin > 70 g/L, ANC > 0.5×10^9^/L, and platelet count > 20×10^9^/L. Overall response (OR) was defined as CR plus PR. No response (NR) was defined as not meeting the criteria of PR. Death or loss of follow-up within 6 months was classified as NR. The time to first response was defined as the interval between ATG initiation and PR. Time to CR was defined as the interval between ATG initiation and CR.

***Statistical analysis***

SPSS software (version 22.0) (IBM Corp., Armonk, NY, USA) was used for statistical analysis. The χ^2^ test was used to analyse categorical variables, and the Mann–Whitney U test was used to analyse continuous variables. The CR and OR rates were compared using the Mantel–Haenszel pooled odds ratio. Time to first response and CR were estimated using the competing risk model. Statistical significance was set at a *P* value < 0.05.

***Predicting factors for haematologic response***

In univariate analysis, no factor was associated with a haematologic response at 3 months. However, disease severity was the only factor associated with a haematologic response at 6 months in the HPAG group (Table S2). The absolute reticulocyte and neutrophil counts, as well as disease severity were the only three factors associated with an overall haematologic response at 3 and 6 months in patients with AA (Table S3). In the multivariate analysis, no independent factor was associated with a haematologic response in patients with AA.

In addition, patients in the HPAG group had a higher probability of CR at 3 and 6 months. No factor was associated with a CR at 3 and 6 months in the HPAG group. In contrast, HPAG plus IST was the only factor associated with a CR at 3 and 6 months in patients with AA (Tables S4 and S5).

| **Table S1. Haematologic response according to severity of aplastic anemia** | | | | | | | |
| --- | --- | --- | --- | --- | --- | --- | --- |
|  | **At 3 months** | | |  | **At 6 months** | | |
|  | Hetrombopag | Control | *P*-value |  | Hetrombopag | Control | *P*-value |
| **Patients with SAA, no.** | 21 | 60 |  |  | 21 | 60 |  |
| HR, no. (%) | 12 (57.1) | 30 (50.0) | 0.575 |  | 17 (80.9) | 35 (58.3) | 0.064 |
| CR, no. (%) | 5 (23.8) | 4 (6.7) | 0.033 |  | 9 (42.9) | 10 (16.7) | 0.015 |
| **Patients with VSAA, no.** | 11 | 36 |  |  | 11 | 36 |  |
| HR, no. (%) | 3 (27.2) | 6 (16.7) | 0.439 |  | 5 (45.5) | 13 (36.1) | 0.581 |
| CR, no. (%) | 2 (18.1) | 1 (2.8) | 0.070 |  | 2 (18.1) | 4 (11.1) | 0.543 |
| Hetrombopag: IST+hetrombopag; Control: IST alone. | | | | | | | |

**Figure S1**

**
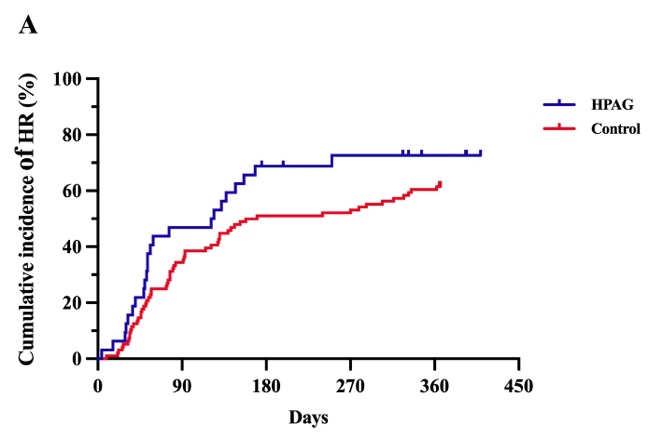

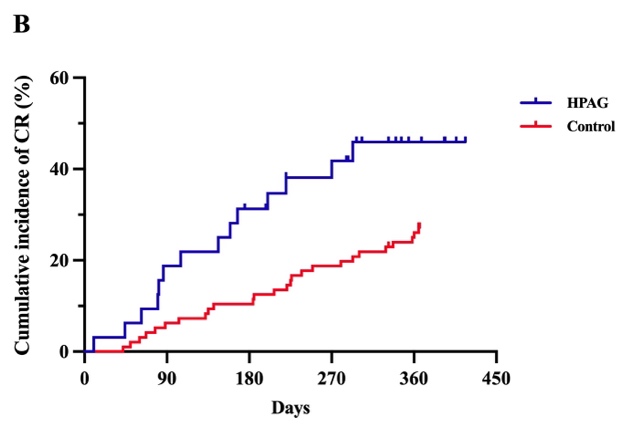
**

| **Table S2. Univariate analysis of hematologic response in the hetrombopag group** | | | | | | | |
| --- | --- | --- | --- | --- | --- | --- | --- |
|  | At 3 months | | |  | At 6 months | | |
|  | Response | Non-response | *P*-value |  | Response | Non-response | *P*-value |
| Numbers of patients | 15 | 17 |  |  | 22 | 10 |  |
| Age (years, median, range) | 33 (16-66) | 49 (13-69) | 0.571 |  | 47 (14-69) | 29 (13-67) | 0.464 |
| Gender (male/female) | 7/8 | 10/7 | 0.498 |  | 12/10 | 5/5 | 0.814 |
| Severe/ Very severe | 12/3 | 9/8 | 0.113 |  | 17/5 | 4/6 | 0.043 |
| Complete blood count (median, range) |  |  |  |  |  |  |  |
| Reticulocyte count (×10^9^/L) | 21.2 (0.6-68.4) | 8.6 (0.2-55.1) | 0.113 |  | 17.2 (0.2- 68.4) | 13.9 (2.4-55.1) | 0.699 |
| Neutrophil count (×10^9^/L) | 0.45 (0.01-1.04) | 0.21 (0-0.65) | 0.016 |  | 0.38 (0-1.04) | 0.20 (0.02-0.65) | 0.281 |
| Platelet count (×10^9^/L) | 10 (1-21) | 6 (1-17) | 0.088 |  | 7 (1-21) | 7 (2-17) | 0.951 |
| Hemoglobin level (g/L) | 57 (34-99) | 49 (39-78) | 0.135 |  | 57 (34-99) | 52 (39-67) | 0.200 |
| PNH clones (+), n (%) | 3 | 8 | 0.063 |  | 7 | 4 | 0.657 |
|  | | | | | | | |

| **Table S3. Univariate analysis of hematologic response in patients with aplastic anemia** | | | | | | | |
| --- | --- | --- | --- | --- | --- | --- | --- |
|  | At 3 months | | |  | At 6 months | | |
|  | Response | Non-response | *P*-value |  | Response | Non-response | *P*-value |
| Numbers of patients | 51 | 77 |  |  | 70 | 58 |  |
| Age (years, median, range) | 32 (9-68) | 49 (7-70) | 0.081 |  | 33 (9-69) | 50 (7-70) | 0.057 |
| Gender (male/female) | 29/22 | 40/37 | 0.586 |  | 41/29 | 28/30 | 0.247 |
| Severe/ Very severe | 42/9 | 39/38 | 0.000 |  | 52/18 | 29/29 | 0.005 |
| Complete blood count (median, range) |  |  |  |  |  |  |  |
| Reticulocyte count (×10^9^/L) | 22.6 (0.6-68.4) | 7.2 (0-63.7) | 0.000 |  | 20.8 (0.2- 68.4) | 7.6 (0-63.7) | 0.007 |
| Neutrophil count (×10^9^/L) | 0.45 (0.01-1.04) | 0.21 (0-1.12) | 0.000 |  | 0.42 (0-1.04) | 0.24 (0-1.12) | 0.007 |
| Platelet count (×10^9^/L) | 8 (1-31) | 7 (0-19) | 0.126 |  | 7 (1-31) | 8 (0-23) | 0.714 |
| Hemoglobin level (g/L) | 61 (33-99) | 57 (36-78) | 0.096 |  | 61 (33-99) | 57 (36-75) | 0.219 |
| PNH clones (+), n (%) | 16 | 21 | 0.618 |  | 22 | 15 | 0.491 |
| Hetrombopag (yes/no) | 15/36 | 17/60 | 0.350 |  | 22/48 | 10/48 | 0.066 |

| **Table S4. Univariate analysis of complete response in the hetrombopag group** | | | | | | | |
| --- | --- | --- | --- | --- | --- | --- | --- |
|  | At 3 months | | |  | At 6 months | | |
|  | CR | Non-CR | *P*-value |  | CR | Non-CR | *P*-value |
| Numbers of patients | 7 | 25 |  |  | 11 | 21 |  |
| Age (years, median, range) | 33 (17-66) | 45 (13-69) | 0.837 |  | 43 (17-66) | 45 (13-69) | 0.662 |
| Gender (male/female) | 3/4 | 14/11 | 0.544 |  | 5/6 | 12/9 | 0.536 |
| Severe/ Very severe | 5/2 | 16/9 | 0.719 |  | 9/2 | 12/9 | 0.169 |
| Complete blood count (median, range) |  |  |  |  |  |  |  |
| Reticulocyte count (×10^9^/L) | 20.7 (0.6-36.1) | 14.8 (0.2-68.4) | 0.983 |  | 21.2 (0.6-46.0) | 12.2 (0.2-68.4) | 0.284 |
| Neutrophil count (×10^9^/L) | 0.45 (0.01-1.04) | 0.29 (0-0.84) | 0.274 |  | 0.45 (0.01-1.04) | 0.24 (0-0.84) | 0.074 |
| Platelet count (×10^9^/L) | 8 (1-21) | 7 (1-17) | 0.631 |  | 10 (1-21) | 6 (1-17) | 0.080 |
| Hemoglobin level (g/L) | 69 (34-81) | 54 (30-99) | 0.264 |  | 66 (34-99) | 49 (39-78) | 0.103 |
| PNH clones (+), n (%) | 1 | 10 | 0.213 |  | 2 | 9 | 0.169 |
|  | | | | | | | |
| **Table S5. Univariate analysis of complete response in patients with aplastic anemia** | | | | | | | |
|  | At 3 months | | |  | At 6 months | | |
|  | CR | Non-CR | *P*-value |  | CR | Non-CR | *P*-value |
| Numbers of patients | 12 | 116 |  |  | 25 | 103 |  |
| Age (years, median, range) | 32 (14-66) | 45 (7-70) | 0.772 |  | 31 (12-66) | 48 (7-70) | 0.115 |
| Gender (male/female) | 8/4 | 61/55 | 0.586 |  | 16/9 | 53/50 | 0.261 |
| Severe/ Very severe | 9/3 | 72/44 | 0.378 |  | 19/6 | 62/41 | 0.143 |
| Complete blood count (median, range) |  |  |  |  |  |  |  |
| Reticulocyte count (×10^9^/L) | 20.9 (0.6-36.3) | 13.4 (0-68.4) | 0.526 |  | 20.9 (0.6- 53.1) | 12.2 (0-68.4) | 0.166 |
| Neutrophil count (×10^9^/L) | 0.44 (0.01-1.04) | 0.32 (0-1.12) | 0.297 |  | 0.43 (0.01-1.04) | 0.30 (0-1.12) | 0.151 |
| Platelet count (×10^9^/L) | 8 (1-21) | 7 (0-31) | 0.902 |  | 8 (1-21) | 7 (0-31) | 0.645 |
| Hemoglobin level (g/L) | 73 (34-96) | 59 (33-99) | 0.079 |  | 62 (34-99) | 58 (33-78) | 0.086 |
| PNH clones (+), n (%) | 3 | 34 | 0.755 |  | 7 | 30 | 0.912 |
| Hetrombopag (yes/no) | 7/5 | 25/91 | 0.005 |  | 11/14 | 21/82 | 0.015 |
|  | | | | | | | |
